# Compliance with early postoperative ambulation and Its associated barriers in hepatobiliary surgery patients within an enhanced recovery after surgery framework

**DOI:** 10.3389/fsurg.2026.1874831

**Published:** 2026-06-24

**Authors:** Yan Zhang, Jingyan Gu, Jie Song, Yun Pan

**Affiliations:** 1 Department of Hepatobiliary Surgery, Affiliated Hospital of Jiangnan University, Wuxi, China; 2 Department of General Surgery, Affiliated Hospital of Jiangnan University, Wuxi, China

**Keywords:** barriers, compliance, early ambulation, enhanced recovery after surgery, hepatobiliary surgery, retrospective study

## Abstract

**Background:**

Early postoperative ambulation is a core element of the Enhanced Recovery After Surgery (ERAS) pathway for hepatobiliary surgery, yet real-world compliance has been reported to be variable and the patient-level barriers in this surgical population remain incompletely characterised. We therefore retrospectively reviewed the routinely collected ERAS quality-control archive of our hepatobiliary surgical unit to describe the actual compliance status and to identify factors associated with non-compliance.

**Methods:**

We retrospectively extracted the records of 320 consecutive adult patients who had undergone elective hepatobiliary surgery between January 2022 and December 2024 and whose perioperative care had followed the institutional ERAS clinical pathway as standard practice. The institutional pathway comprised eight standardised components organised across the preoperative, intraoperative and postoperative phases, including structured patient education, carbohydrate loading, goal-directed fluid therapy, multimodal opioid-sparing analgesia, restrictive abdominal drainage, omission of routine nasogastric tubes, early oral feeding, and the early-ambulation prescription. Compliance with the early ambulation protocol [out-of-bed activity by postoperative day (POD) 1 and ambulation distance ≥30 m by POD 2] and patient-reported barriers documented in the routine nursing-assessment questionnaire were retrieved from the electronic medical record. The primary outcome was compliance status, while postoperative length of stay, time to first flatus, pulmonary complications, surgical site infection, symptomatic venous thromboembolism, major postoperative haemorrhage, in-hospital all-cause mortality and 30-day readmission were pre-specified secondary outcomes. Group allocation (compliant vs. non-compliant) reflected real-world clinical and patient/family decisions, not investigator-led randomisation. Univariable comparisons (chi-square, Fisher exact for low-frequency events, or Welch t-test as appropriate) and multivariable logistic regression adjusted for age, sex, BMI and the pre-specified clinical predictors were used to identify factors associated with non-compliance.

**Results:**

Two hundred and three of 320 patients (63.4%) were classified as compliant with the early ambulation protocol. Compliance varied markedly by procedure, ranging from 80.0% after laparoscopic hepatectomy to 33.3% after biliary reconstruction. The most frequently documented patient-reported barriers were postoperative pain (46.3%), fatigue/weakness (37.0%), knowledge deficit (35.6%), and the presence of multiple drainage tubes (35.0%). In multivariable analysis, open or major hepatobiliary surgery [adjusted odds ratio (aOR) 3.53, 95% CI 1.92–6.48], having ≥3 drainage tubes (aOR 2.52, 1.43–4.42), Clinical Frailty Scale score ≥4 (aOR 2.51, 1.45–4.34), age ≥65 years (aOR 2.30, 1.26–4.18), and absence of a documented family companion (aOR 1.80, 1.03–3.13) were all independently associated with non-compliance. The model demonstrated acceptable discrimination (Hosmer–Lemeshow *χ*^2^ = 6.41, *P* = 0.602; area under the ROC curve 0.812, 95% CI 0.765–0.859) without evidence of problematic multicollinearity (all variance inflation factors <1.8). Non-compliance was associated with longer hospital stay (11.9 ± 3.2 vs. 7.5 ± 2.3 days, *P* < 0.001) and higher rates of pulmonary complications (23.9% vs. 7.4%, *P* < 0.001) and 30-day readmission (14.5% vs. 3.4%, *P* = 0.001).

**Conclusions:**

In this retrospective single-centre cohort, just under two-thirds of hepatobiliary surgery patients were compliant with the early ambulation component of the ERAS pathway. Compliance was associated with surgical magnitude, drainage burden, frailty, age, and family companionship, and may be related to the higher rate of postoperative complications observed in non-compliant patients. These findings suggest that targeted, multifactorial barrier-mitigation strategies could improve early-ambulation adherence in hepatobiliary surgery, although the observational design does not permit causal inference.

## 1 Introduction

Hepatobiliary surgery — including hepatectomy, complex cholecystectomy with common-bile-duct exploration, biliary reconstructions, and pancreaticoduodenectomy — remains a high-stress, high-morbidity intervention, particularly in older or comorbid patients. Over the past two decades, the Enhanced Recovery After Surgery (ERAS) pathway has been progressively adopted in hepatobiliary units worldwide, with growing evidence that adherence to its multimodal perioperative components is associated with shorter length of stay, fewer complications, and lower cost compared with traditional perioperative care (1, 2). Within the ERAS bundle, early postoperative ambulation is repeatedly highlighted as a low-cost, high-impact element that counteracts the deleterious physiological effects of bed rest (3).

Although the ERAS bundle is intentionally multimodal, and the synergistic effect of the components as a whole is well documented (1, 2), early ambulation is in many respects unique. First, unlike most other components which are largely delivered by clinicians (analgesia, fluid management, drain rationalisation, anaesthetic technique), ambulation requires active patient participation and is therefore the component most sensitive to patient-level barriers, motivation and the bedside care environment. Second, recent audits have consistently reported that early ambulation is among the ERAS items with the widest variation between protocol and real-world performance (4, 5). Third, an isolated, granular characterisation of early-ambulation compliance and its modifiable barriers has direct implications for nurse-led quality-improvement initiatives, which would not be visible in a global ERAS-compliance metric averaged across all items. We therefore deliberately focused on this single domain in the present analysis, while still describing the full ERAS bundle implemented at our unit (Section 2.2) to allow the findings to be interpreted in pathway context.

The 2022 ERAS Society guideline for liver surgery explicitly recommends that out-of-bed activity be initiated on the day of surgery and continued daily until discharge, although the optimal target distance and duration are not specified (2). A recent systematic review further reported that protocols starting ambulation within the early postoperative period are associated with improved recovery outcomes and shorter hospitalisation after gastrointestinal surgery (4). In hepatopancreatobiliary (HPB) units in particular, however, real-world compliance has been reported to be markedly lower than what trial protocols achieve, with audited rates often below 30%–40% in some care domains and a substantial proportion of patients failing to achieve a 30-metre ambulation target by POD 2 (5, 6).

Why hepatobiliary patients in particular struggle with early mobilisation is multifactorial. Reported barriers cluster around (i) symptoms (incisional and visceral pain, dizziness, nausea, fatigue), (ii) the surgical apparatus itself [multiple abdominal drains, biliary tubes, urinary catheter, intravenous lines and patient-controlled analgesia (PCA) pumps], (iii) cognitive and emotional factors (knowledge deficit about the benefits of ambulation, fear of wound dehiscence or bleeding, anxiety and depression), and (iv) social and organisational factors (insufficient family companionship, ward staffing, lack of standardised mobilisation protocols) (5–7). Compliance has consistently been lower in patients undergoing major or open surgery and in older or frail patients (5, 6, 8).

Although several recent studies have examined ERAS compliance overall in liver surgery (8–10) or have surveyed healthcare providers on ERAS implementation (11, 12), comparatively little contemporary evidence has been generated specifically on the patient-reported barriers to early ambulation within a hepatobiliary cohort, and almost all available data have been collected prospectively in tertiary, research-oriented centres. By retrospective interrogation of routinely collected ERAS quality-control data we refer to the structured, second-pass analytic use of variables that had already been entered prospectively into the institutional ERAS quality-control registry and the perioperative nursing archive as part of routine pathway audit. No additional case-finding, no additional questionnaire administration, and no investigator-driven follow-up visit were performed; the analysis was confined to the audit dataset as it had been assembled in real time at the bedside. From a quality-improvement standpoint, this approach to the routinely collected data and the structured barrier questionnaire that is administered as part of standard nursing care can offer an unselected, real-world view of compliance and may help identify modifiable factors that are amenable to local pathway redesign.

Accordingly, the present study had two objectives. First, we sought to describe the proportion of hepatobiliary surgical patients in our unit who were compliant with the early ambulation component of the institutional ERAS pathway and to characterise the time course and quantity of their postoperative ambulation. Second, we sought to identify the patient-, procedure- and care-level factors that were associated with non-compliance, and to explore whether non-compliance, as observed in routine practice, was associated with adverse short-term postoperative outcomes.

## 2 Materials and methods

### 2.1 Study design and ethical considerations

This was a single-centre retrospective study conducted at the Department of Hepatobiliary Surgery of a tertiary referral teaching hospital. The study used data already accumulated in the institutional ERAS quality-control registry, the perioperative nursing assessment archive, and the hospital electronic medical record system, all of which had been generated as part of routine clinical care during the study period. No prospective intervention, additional questionnaire administration, additional patient selection, or extra follow-up visit was performed for the purposes of this study; all variables analysed were those already documented prospectively during the index admission as part of routine care and quality-control recording. The study protocol was reviewed and approved by the Institutional Ethics Committee of the Affiliated Hospital of Jiangnan University. Because the analysis used de-identified routine clinical and quality-control data, and because no patient contact, additional questionnaire administration, or change to clinical care was undertaken for the purposes of the study, the committee formally waived the requirement for individual written informed consent, in accordance with the national regulations on ethical review of human biomedical research issued by the Chinese National Health Commission. The same ethics and consent wording is reflected consistently in the Abstract and the Ethics declaration at the end of the manuscript.

### 2.2 Setting and routine clinical pathway

Since 2021, the department has operated a unit-wide ERAS clinical pathway for elective hepatobiliary surgery, structured according to the 2022 ERAS Society recommendations for liver surgery (2) and adapted locally for biliary and pancreaticobiliary procedures. The pathway comprises eight standardised components organised across the perioperative continuum. In the preoperative phase, two components are delivered: structured patient and family education by trained ward nurses 24–48 h before surgery, and oral carbohydrate loading up to two hours before induction. In the intraoperative phase, one component is delivered: standardised goal-directed fluid therapy, applied under a unit-wide opioid-sparing anaesthetic protocol; this anaesthetic protocol forms the upstream half of the perioperative multimodal opioid-sparing analgesia component, which is counted only once and is reported under the postoperative phase below to avoid double-counting. Minimally invasive (laparoscopic) approaches are not a stand-alone ERAS component in our institutional pathway but are offered when oncologically and technically appropriate, with the actual rate of laparoscopic resection captured as a procedure-level variable in the present analysis (Section 2.5.2). In the postoperative phase, five components are delivered: multimodal opioid-sparing analgesia (surgical-site infiltration with 0.375% ropivacaine 20 mL at closure, scheduled intravenous flurbiprofen axetil 50 mg every 12 h and oral acetaminophen 0.5 g every 8 h, with optional fentanyl-based intravenous PCA as a step-up; single-shot transversus-abdominis-plane blocks were used selectively in laparoscopic cases at the discretion of the anaesthetist); omission of routine nasogastric tubes; restrictive abdominal drainage when oncologically appropriate; early oral feeding from the day of surgery; and the standardised early-ambulation prescription. The omission of selected components of the published ERAS Society liver-surgery bundle — such as routine epidural analgesia and routine immunonutrition — reflects pragmatic local adaptation to our case-mix, anaesthetic culture and resource availability, and these omissions were made at the time of pathway design and not for the purposes of the present analysis. The early-ambulation order is written by the surgical team on POD 0 and is supervised by the bedside nurse and, when needed, by the ward physiotherapist. The pathway and its quality indicators have been routinely audited monthly via the institutional ERAS quality-control dashboard.

### 2.3 Patient identification and eligibility

Eligible patients were identified retrospectively by querying the hospital information system for all consecutive adults aged ≥18 years who had undergone elective hepatobiliary surgery between 1 January 2022 and 31 December 2024 and whose perioperative care had been documented under the institutional ERAS pathway. Surgical procedures included open or laparoscopic hepatectomy, laparoscopic cholecystectomy combined with common-bile-duct exploration, biliary reconstruction (hepaticojejunostomy or choledochojejunostomy), and pancreaticoduodenectomy.

Inclusion criteria were as follows: (1) age ≥18 years at the time of surgery; (2) elective hepatobiliary surgery during the study window; (3) full enrolment on the institutional ERAS clinical pathway from preoperative admission until discharge; and (4) availability of a complete electronic record covering operative parameters, daily nursing-assessment notes, and the structured early-ambulation barrier questionnaire.

Exclusion criteria were as follows: (1) emergency or unplanned surgery, since the ERAS pathway and its preoperative education component had not been applied; (2) postoperative intensive-care unit (ICU) stay >48 h, severe haemodynamic instability, ongoing mechanical ventilation, or another medical contraindication to ambulation as documented by the treating physician; (3) reoperation during the index admission; (4) neurological, orthopaedic, or musculoskeletal disability that had impaired baseline ambulation before surgery; and (5) incomplete records or missing barrier-questionnaire data.

A total of 412 consecutive patients undergoing elective hepatobiliary surgery on the institutional ERAS pathway were identified during the three-year window. After applying the exclusion criteria, 41 patients were excluded for postoperative ICU stay >48 h or other documented medical contraindication to ambulation, 14 for reoperation during the index admission, 12 for pre-existing neurological, orthopaedic or musculoskeletal disability, and 25 for incomplete records or missing items in the barrier questionnaire, leaving 320 patients with complete data who were retained for analysis. Missing data within retained patients were minimal (<1.5% for any single variable) and were not imputed; complete-case analysis was applied throughout.

### 2.4 Group allocation

Group allocation was performed retrospectively, on the basis of the clinical events already recorded in the chart, and reflected real-world bedside decisions made by patients, families and the multidisciplinary team rather than any investigator-led randomisation. Following the institutional standard, patients were classified as compliant with the early ambulation protocol if both of the following criteria, recorded by the bedside nurse on the daily mobilisation flow-sheet, were met: (1) successful out-of-bed activity (defined as sitting on the edge of the bed for at least 15 min or walking at the bedside) was achieved by 24 h after the end of surgery (POD 1); and (2) cumulative ambulation distance on POD 2 was at least 30 metres, an institutional threshold that has been adopted from the published HPB ERAS literature (5, 6). Although the 30-metre target on POD 2 is necessarily a pragmatic and somewhat arbitrary cut-off, it was selected for institutional use because it represents the distance between the patient room and the nursing station on our ward, can be measured by the bedside nurse without specialist equipment, and is congruent with the targets reported in two recent HPB-ERAS quality-improvement programmes (5, 6); in a sensitivity exploration around this threshold (Section 3.6) the overall pattern of associations was preserved. Patients who failed to meet either criterion were classified as non-compliant. Because group assignment was driven entirely by the patient's actual clinical course, the groups were inherently non-randomised, and inter-group baseline differences were anticipated and addressed analytically (Section 2.7).

### 2.5 Variables extracted

#### 2.5.1 Demographic and clinical characteristics

Demographic and baseline clinical variables were extracted from the admission record and included age, sex, body-mass index, American Society of Anesthesiologists (ASA) physical status, the Clinical Frailty Scale (CFS) score routinely assigned at admission by the ward nurse for patients aged ≥60 years (and recorded as the lowest level for younger patients on the basis of structured questioning), and pre-existing comorbidities (diabetes mellitus, hypertension, cirrhosis confirmed by imaging or histology). Preoperative documentation of structured ERAS education and the presence of a family companion during the postoperative period were also retrieved as routinely recorded yes/no fields on the standardised nursing checklist.

#### 2.5.2 Operative and perioperative variables

Operative data were extracted from the anaesthesia record and the operative report, and included surgery type, operative approach (open vs. laparoscopic/minimally invasive), the operating surgeon's years of independent hepatobiliary experience (dichotomised at ≥10 years), operative duration, estimated blood loss, and the number of intra-abdominal drainage tubes (peritoneal, hepatic-bed, biliary, and Foley catheter for the purposes of mobilisation burden). Postoperative variables included the highest numerical rating scale (NRS) pain score on POD 1, recorded routinely by nursing staff every 4 h, the type of postoperative analgesic regimen actually delivered (multimodal opioid-sparing alone vs. multimodal plus PCA, with or without wound infiltration or a single-shot transversus-abdominis-plane block), and the use of a patient-controlled analgesia (PCA) device.

#### 2.5.3 Ambulation parameters

Ambulation outcomes were extracted from the standardised nursing mobilisation flow-sheet that is completed for every patient on the ERAS pathway. The retrieved variables included (1) the number of hours from the end of surgery to the first documented out-of-bed activity, (2) the cumulative ambulation distance achieved on POD 2 measured in metres along the marked ward corridor, and (3) the daily ambulation status (yes/no) for POD 0 through POD 3.

#### 2.5.4 Barrier questionnaire

As part of routine ERAS quality-control, the bedside nurse administers a 10-item structured early-ambulation barrier questionnaire to every patient on POD 1 and POD 2 and documents whether each potential factor prevented or limited the prescribed ambulation, regardless of whether the day's ambulation was eventually completed; this design allows the questionnaire to record concurrent perceived barriers in both groups rather than only retrospective justifications in patients who failed to mobilise. The instrument was developed in 2020 by a working group of senior ward nurses, ERAS coordinators and a surgical methodologist, on the basis of the published HPB-ERAS and gastrointestinal-surgery barrier literature (5–7) and the conceptual barrier framework proposed by Tazreean and colleagues (3); an initial 13-item draft was piloted on 30 consecutive hepatobiliary patients during the first quarter of 2021, three semantically overlapping items were dropped, and three items were re-worded for clarity, producing the 10-item version used unchanged across the unit since mid-2021. Bedside nurses receive standardised training on questionnaire administration during their unit induction and at yearly refresher sessions; inter-rater consistency in a quality-control sub-sample of 40 paired patient-nurse administrations was substantial (Cohen's *κ* = 0.81). The full Chinese-language form and English translation are provided in the Supplementary Material. We acknowledge that, despite this developmental and training process, the instrument has not undergone formal psychometric validation in a separate cohort, and this is restated as a limitation (Section 4.1). It captures the presence (yes/no) of the following potential barriers: (1) postoperative pain (NRS >= 4 at the time of the planned ambulation), (2) fatigue or generalised weakness, (3) presence of multiple drainage tubes (>= 3 tubes/lines tethered to the patient), (4) fear of wound dehiscence or postoperative bleeding, (5) dizziness on standing, (6) nausea or vomiting, (7) intravenous infusion line or PCA pump perceived as a tether, (8) knowledge deficit or unawareness of the benefit of early ambulation, (9) anxiety or depressive symptoms, and (10) inadequate family companionship at the time of the planned mobilisation. For the present analysis, a barrier was considered present if it had been documented at least once during the POD 1–2 window.

#### 2.5.5 Outcomes

The primary outcome was compliance status with the early ambulation protocol (compliant vs. non-compliant). Pre-specified secondary outcomes, retrieved from the electronic medical record, were total postoperative length of hospital stay (in days), time to first flatus (in hours from end of surgery), and the occurrence within the index admission of (1) pulmonary complication (clinically diagnosed pneumonia and/or radiologically confirmed atelectasis requiring active treatment), (2) surgical site infection per Centers for Disease Control and Prevention criteria, (3) major postoperative haemorrhage requiring transfusion of ≥2 units of packed red cells or invasive intervention, (4) in-hospital all-cause mortality, and (5) symptomatic venous thromboembolism confirmed by ultrasonography or computed tomography. Thirty-day all-cause readmission was retrieved from the hospital discharge follow-up registry.

### 2.6 Sample size considerations

Because all consecutive eligible patients during the three-year window were included, no prior power calculation was performed. With 117 non-compliance events observed in the 320-patient cohort, the analysis was considered adequate for a multivariable logistic-regression model with approximately 10 covariates under conventional events-per-variable considerations.

### 2.7 Statistical analysis

Continuous variables were summarised as mean ± standard deviation and were compared between the compliant and non-compliant groups by the independent-samples Welch t-test, after assessment of normality with the Shapiro–Wilk test. Categorical variables were summarised as numbers and percentages and were compared by the chi-square test or Fisher's exact test as appropriate; specifically, Fisher's exact test (scipy.stats.fisher_exact, two-sided) was used whenever any expected cell count was less than 5, which applied to in-hospital all-cause mortality, major postoperative haemorrhage and symptomatic venous thromboembolism in the present cohort, while the chi-square test (without continuity correction) was used for all other categorical comparisons. The prevalence of each barrier item was reported overall and stratified by compliance group.

A multivariable logistic-regression model was fitted with non-compliance as the dependent variable and the following pre-specified candidate predictors as independent variables: age ≥65 years [chosen because in our setting the institutional CFS is routinely scored only from age ≥60 years and because 65 years is the threshold most commonly used in geriatric surgical risk-stratification (13, 14)], open or major hepatobiliary surgery (open hepatectomy, pancreaticoduodenectomy, or biliary reconstruction), POD 1 NRS pain ≥4 [because NRS ≥4 is the clinically accepted cut-off for inadequately controlled moderate pain (15)], ≥3 drainage tubes (reflecting the institutional definition of high tube burden, three corresponding to a patient typically tethered by a peritoneal drain, biliary stent and urinary catheter), CFS ≥4 [corresponding to the published cut-off for clinically meaningful frailty (13)], absence of preoperative ERAS education, absence of a documented family companion, and ASA class ≥3. Predictors were intentionally dichotomised to mirror the clinical thresholds used in routine bedside decision-making and to facilitate translation of the model into a pragmatic risk-flag at admission; in addition, continuous forms of age, CFS, drain count and NRS pain were each entered into separate sensitivity logistic models and the assumption of linearity in the logit was visually checked with restricted cubic splines (3 knots), which did not suggest material departure from linearity for any of the four variables. Predictors fell into two conceptual layers: structural/baseline factors (age, frailty, type of surgery, drain count, ASA class, family companion, preoperative education), which are largely fixed at admission, and symptom-level factors (POD 1 pain, fatigue, dizziness, nausea), which co-evolve with the patient's postoperative course. POD 1 pain in particular was retained in the model as a clinically modifiable predictor but is acknowledged to lie on the same causal pathway as ambulation and may therefore act partly as a mediator rather than a strictly upstream cause; this is discussed in Section 4. Model fit was assessed with the Hosmer–Lemeshow goodness-of-fit test (*P* > 0.05 indicating adequate fit) and discrimination with the area under the receiver-operating-characteristic curve (AUROC). Multicollinearity was assessed by the variance inflation factor (VIF), with VIF > 5 considered problematic. Demographic variables (age, sex and BMI) were retained in the multivariable model irrespective of their univariable P-value to control for residual confounding by basic demographics. Adjusted odds ratios with 95% confidence intervals were reported. Two-sided *P* values < 0.05 were considered statistically significant. To reduce confounding inherent to the non-randomised design, all regression analyses were adjusted for the variables listed above; nevertheless, residual confounding by unmeasured variables (including provider behaviour and patient motivation) is acknowledged in the discussion. All analyses were performed in Python v3.11 using the pandas, scipy and statsmodels libraries; figures were produced with matplotlib and seaborn.

## 3 Results

### 3.1 Cohort characteristics

After application of the inclusion and exclusion criteria, 320 patients with complete records were retained for analysis. Of these, 203 (63.4%) were classified as compliant and 117 (36.6%) as non-compliant with the early ambulation protocol. Baseline demographic, clinical and operative characteristics are presented in Table 1. Patients in the non-compliant group were on average older (61.5 ± 11.3 vs. 56.4 ± 10.7 years, *P* < 0.001), had higher CFS scores (3.5 ± 1.3 vs. 3.0 ± 1.2, *P* = 0.001), reported higher POD 1 NRS pain scores (5.4 ± 1.9 vs. 4.1 ± 1.7, *P* < 0.001), had marginally longer operations (229.6 vs. 213.3 min, *P* = 0.037) and tended to be less frequently accompanied by a family member during ward stay (62.4% vs. 72.9%, *P* = 0.067). Open or major hepatobiliary surgery (open hepatectomy, pancreaticoduodenectomy or biliary reconstruction combined) was undertaken in 82 of 203 compliant patients (40.4%) and in 86 of 117 non-compliant patients (73.5%), corresponding to an overall cumulative frequency of 168/320 (52.5%) in the cohort (*P* < 0.001). The distribution of comorbidities, ASA class, sex and BMI did not differ significantly between groups (all *P* > 0.10).

**Table 1:** Baseline demographic, clinical and operative characteristics of the study cohort, stratified by compliance with the early ambulation protocol.

Variable	Compliant (*n* = 203)	Non-compliant (*n* = 117)	*P* value
Age (years), mean ± SD	56.4 ± 10.7	61.5 ± 11.3	<0.001
Sex, male/female, n	126/77	73/44	1.000
BMI (kg/m^2^), mean ± SD	23.4 ± 2.8	23.3 ± 3.0	0.731
ASA class III–IV, n (%)	64 (31.5)	44 (37.6)	0.273
Diabetes mellitus, n (%)	48 (23.6)	32 (27.4)	0.546
Hypertension, n (%)	63 (31.0)	34 (29.1)	0.807
Cirrhosis, n (%)	46 (22.7)	27 (23.1)	1.000
Clinical Frailty Scale, mean ± SD	3.0 ± 1.2	3.5 ± 1.3	0.001
Surgery type, n (%)			
Laparoscopic hepatectomy	80 (39.4)	20 (17.1)	
Open hepatectomy	47 (23.2)	47 (40.2)	
Lap. cholecystectomy + CBD exploration	41 (20.2)	11 (9.4)	
Pancreaticoduodenectomy	24 (11.8)	17 (14.5)	
Biliary reconstruction	11 (5.4)	22 (18.8)	<0.001
Open or major HPB surgery, n (%)	82 (40.4)	86 (73.5)	<0.001
Minimally invasive (laparoscopic) approach, n (%)	121 (59.6)	31 (26.5)	<0.001
Operating surgeon experience ≥10 y, n (%)	156 (76.8)	82 (70.1)	0.198
Operation time (min), mean ± SD	213.3 ± 73.2	229.6 ± 62.8	0.037
Estimated blood loss (mL), mean ± SD	282 ± 188	316 ± 203	0.145
Number of drains, mean ± SD	2.1 ± 0.9	2.3 ± 0.9	0.049
POD 1 NRS pain score, mean ± SD	4.1 ± 1.7	5.4 ± 1.9	<0.001
PCA used, n (%)	132 (65.0)	69 (59.0)	0.338
Preoperative ERAS education, n (%)	161 (79.3)	84 (71.8)	0.164
Family companion documented, n (%)	148 (72.9)	73 (62.4)	0.067
Analgesic regimen actually delivered, n (%)			
Multimodal opioid-sparing only	71 (35.0)	48 (41.0)	
Multimodal + PCA	132 (65.0)	69 (59.0)	
+Wound infiltration	188 (92.6)	104 (88.9)	
+Single-shot TAP block	47 (23.2)	12 (10.3)	0.004

ASA, American Society of Anesthesiologists; BMI, body-mass index; CBD, common bile duct; ERAS, Enhanced Recovery After Surgery; HPB, hepatopancreaticobiliary; NRS, numerical rating scale; PCA, patient-controlled analgesia; POD, postoperative day; SD, standard deviation; TAP, transversus abdominis plane. *P* values are derived from Welch t-test or chi-square/Fisher exact tests as appropriate.

### 3.2 Compliance pattern and ambulation distance

**Figure 1:**
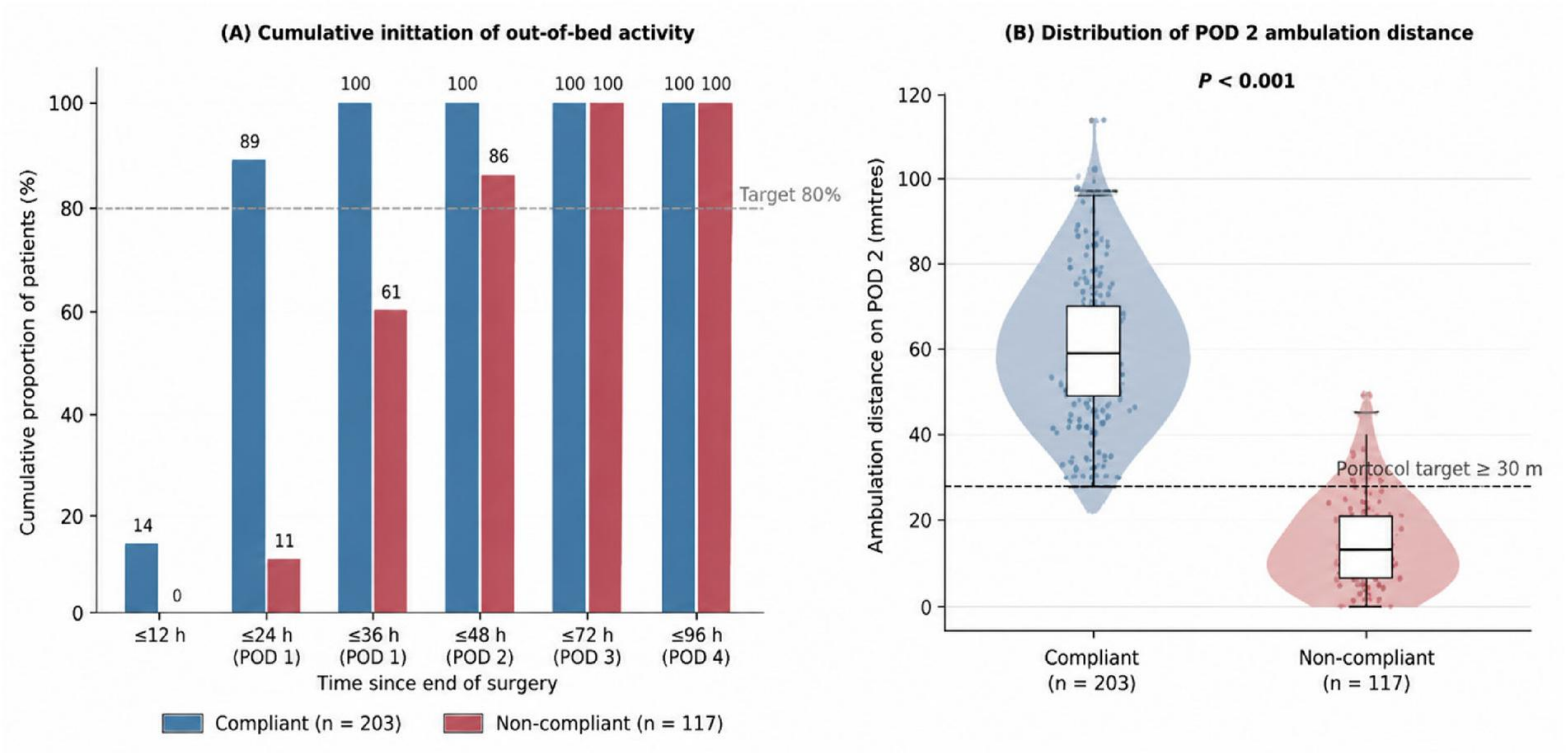
**(A)** cumulative proportion of patients who had achieved their first out-of-bed activity at successive time points after end of surgery, stratified by compliance group; the dotted line indicates the institutional 80% target by 24 h. **(B)** Violin and box plot of cumulative ambulation distance achieved on postoperative day 2; the dashed reference line indicates the protocol target of ≥30 metres.

Figure 1 summarises the cumulative initiation of out-of-bed activity over the first four postoperative days and the distribution of ambulation distance achieved on POD 2. By POD 1, 89% of compliant patients had achieved their first out-of-bed activity, compared with only 11% of non-compliant patients (Figure 1A); by POD 2, the corresponding values were 100% vs. 86%. The institutional 80% target for first out-of-bed activity by POD 1 was therefore met overall in the compliant group but was not approached in the non-compliant group until POD 2.

The distribution of POD 2 ambulation distance is shown in Figure 1B. Compliant patients walked a median of approximately 60 metres (mean 58.7 ± 16.2 m), with most exceeding the 30-metre target by a substantial margin. In contrast, non-compliant patients walked a median of approximately 17 metres (mean 18.2 ± 11.5 m), the great majority of whom remained below the 30-metre threshold (between-group *P* < 0.001). A small fraction of non-compliant patients nevertheless reached a POD 2 distance above 30 metres (visible in the upper tail of the right-hand violin in Figure 1B); on chart review these were uniformly patients who had failed the POD 1 out-of-bed criterion (delayed first mobilisation beyond 24 h after end of surgery) but who, once mobilised, were able to cover more than 30 metres on POD 2. Because compliance was defined as conjunctive (POD 1 AND POD 2 criteria both met), they remained classified as non-compliant despite an adequate POD 2 distance, illustrating that the temporal initiation of mobilisation, not only its eventual quantity, contributes to compliance status. Compliance varied considerably by procedure: 80.0% after laparoscopic hepatectomy, 78.8% after laparoscopic cholecystectomy with common-bile-duct exploration, 58.5% after pancreaticoduodenectomy, 50.0% after open hepatectomy, and 33.3% after biliary reconstruction.

### 3.3 Patient-reported barriers to early ambulation

**Figure 2:**
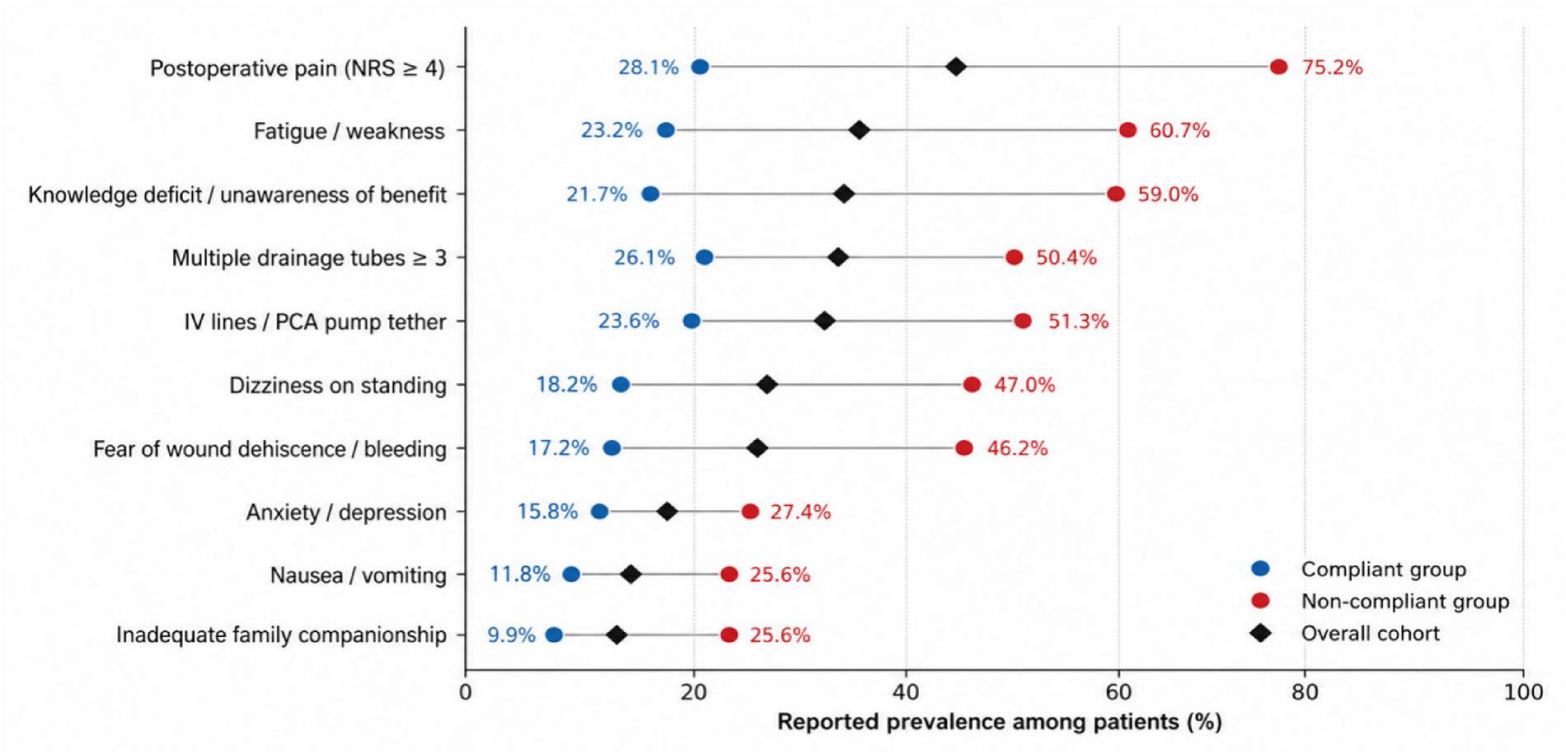
Dumbbell plot of the prevalence of patient-reported barriers to early ambulation, stratified by compliance group. Black diamonds indicate the prevalence in the overall cohort; blue and red circles indicate the prevalence in the compliant and non-compliant groups, respectively.

The prevalence of each documented barrier in the routine nursing questionnaire is shown in Figure 2. Across the whole cohort, the four most frequently endorsed barriers were postoperative pain (46.3%), fatigue or generalised weakness (37.0%), knowledge deficit or unawareness of the benefit of ambulation (35.6%), and the perceived burden of multiple drainage tubes (35.0%). Detailed group-stratified prevalences are presented in Figure 2; to avoid redundancy with the figure, the equivalent tabular summary that had appeared in earlier versions of this work has been removed and the figure is now the single authoritative source for these data.

Every barrier was reported substantially more frequently by patients in the non-compliant group than in the compliant group. The widest absolute gaps were observed for pain (75.2% vs. 28.1%), knowledge deficit (59.8% vs. 21.7%), fatigue (60.7% vs. 23.2%), fear of wound dehiscence or bleeding (46.2% vs. 17.2%), and dizziness on standing (47.0% vs. 18.2%). Multiple drainage tubes and IV/PCA tethering were endorsed by approximately half of non-compliant patients. Inadequate family companionship and nausea/vomiting, although less prevalent overall, were also disproportionately reported in the non-compliant group.

### 3.4 Factors associated with non-compliance

**Figure 3:**
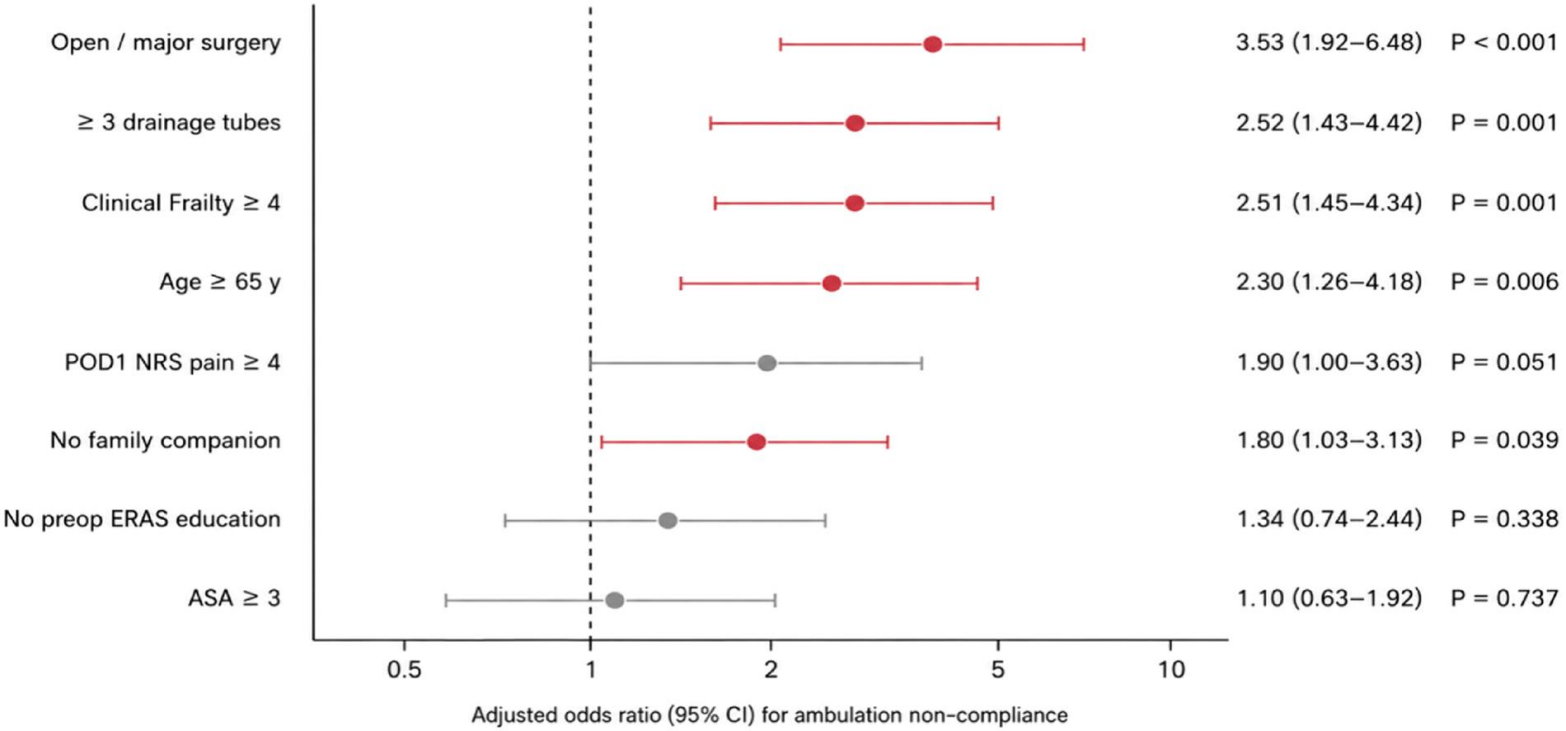
Forest plot of adjusted odds ratios with 95% confidence intervals from the multivariable logistic-regression model identifying factors associated with non-compliance with the early ambulation protocol. Variables in red were statistically significant (*P* < 0.05); the dashed vertical reference line indicates the null value (OR = 1). Marker size is proportional to the odds-ratio point estimate. Controlling variables included in the model: age ≥65 years, sex, body-mass index, open or major hepatobiliary surgery, POD 1 NRS pain ≥4, ≥3 drainage tubes, Clinical Frailty Scale ≥4, absence of preoperative ERAS education, absence of a documented family companion, and ASA class ≥3. Model performance: Hosmer–Lemeshow *P* = 0.602; AUROC 0.812; maximum VIF 1.74.

Multivariable logistic-regression analysis is presented in Figure 3. The model adjusted simultaneously for age ≥65 years, sex, BMI, open or major hepatobiliary surgery, POD 1 NRS pain ≥4, ≥3 drainage tubes, CFS ≥4, absence of preoperative ERAS education, absence of a documented family companion, and ASA class ≥3. The Hosmer–Lemeshow test indicated adequate goodness of fit (*χ*^2^ = 6.41, *P* = 0.602), AUROC was 0.812 (95% CI 0.765–0.859), and the highest variance inflation factor across the included covariates was 1.74, well below the conventional threshold of 5, indicating no problematic collinearity. Five variables were independently associated with greater odds of non-compliance with the early ambulation protocol: open or major hepatobiliary surgery (aOR 3.53, 95% CI 1.92–6.48; *P* < 0.001), the presence of three or more drainage tubes (aOR 2.52, 1.43–4.42; *P* = 0.001), CFS ≥4 (aOR 2.51, 1.45–4.34; *P* = 0.001), age ≥65 years (aOR 2.30, 1.26–4.18; *P* = 0.006), and absence of a documented family companion (aOR 1.80, 1.03–3.13; *P* = 0.039). POD 1 NRS pain ≥4 was associated with a borderline-significant increase in the odds of non-compliance (aOR 1.90, 1.00–3.63; *P* = 0.051). Absence of preoperative ERAS education and ASA class ≥3 were not independently associated with non-compliance after adjustment.

### 3.5 Postoperative outcomes

Postoperative short-term outcomes are summarised in Table 2. Compared with compliant patients, non-compliant patients had a significantly longer postoperative length of stay (11.9 ± 3.2 vs. 7.5 ± 2.3 days, *P* < 0.001), longer time to first flatus (71.4 ± 15.2 vs. 45.7 ± 14.1 h, *P* < 0.001), higher pulmonary complication rate (23.9% vs. 7.4%, *P* < 0.001), higher surgical-site-infection rate (10.3% vs. 3.4%, *P* = 0.025), higher rate of symptomatic venous thromboembolism (6.8% vs. 1.0%, *P* = 0.006 by Fisher's exact test), and higher 30-day readmission rate (14.5% vs. 3.4%, *P* = 0.001). Major postoperative haemorrhage requiring transfusion of ≥2 units of packed red cells or invasive intervention occurred in 4 of 203 compliant patients (2.0%) and in 7 of 117 non-compliant patients (6.0%; *P* = 0.106 by Fisher's exact test). In-hospital all-cause mortality was rare in both groups [1/203 [0.5%] vs. 2/117 [1.7%]; *P* = 0.557 by Fisher's exact test]. In an exploratory analysis adjusting the unadjusted between-group differences in length of stay and the three principal complication endpoints for age, sex, BMI, surgery type and CFS, the association of non-compliance with prolonged length of stay (adjusted mean difference 3.6 days, 95% CI 2.9–4.4) and with pulmonary complications (aOR 2.74, 95% CI 1.34–5.62) remained statistically significant, although attenuated, while the association with 30-day readmission was reduced to borderline significance (aOR 2.95, 95% CI 0.98–8.85). These adjusted analyses are exploratory and are intended only to indicate the direction and approximate magnitude of residual association after accounting for the most obvious confounders; they should not be interpreted as evidence of causality. Because group allocation reflected real-world clinical decisions and was non-random, the differences between groups should be interpreted as associations rather than as evidence of a causal effect of ambulation *per se*.

**Table 2:** Postoperative ambulation parameters and short-term outcomes by compliance group.

Outcome	Compliant (*n* = 203)	Non-compliant (*n* = 117)	*P* value
Time to first out-of-bed (h), mean ± SD	17.6 ± 5.3	35.3 ± 10.3	<0.001
Ambulation distance on POD 2 (m), mean ± SD	58.7 ± 16.2	18.2 ± 11.5	<0.001
Time to first flatus (h), mean ± SD	45.7 ± 14.1	71.4 ± 15.2	<0.001
Postoperative length of stay (d), mean ± SD	7.5 ± 2.3	11.9 ± 3.2	<0.001
Pulmonary complication, n (%)	15 (7.4)	28 (23.9)	<0.001
Surgical site infection, n (%)	7 (3.4)	12 (10.3)	0.025
Symptomatic VTE, n (%)	2 (1.0)	8 (6.8)	0.006^†^
30-day readmission, n (%)	7 (3.4)	17 (14.5)	0.001
Major haemorrhage requiring transfusion/intervention, n (%)	4 (2.0)	7 (6.0)	0.106^†^
In-hospital all-cause mortality, n (%)	1 (0.5)	2 (1.7)	0.557†

POD, postoperative day; SD, standard deviation; VTE, venous thromboembolism.

† *P* value calculated by Fisher's exact test (scipy.stats.fisher_exact, two-sided), used for low-frequency outcomes with any expected cell count <5.

### 3.6 Sensitivity analysis on the ambulation distance threshold

To verify that the 30-metre POD 2 threshold did not unduly drive our findings, we re-classified the cohort using two alternative cut-offs (20 metres and 50 metres) drawn from the wider HPB-ERAS literature (5, 6). Under a 20-metre cut-off, 217 patients (67.8%) were classified as compliant; under a 50-metre cut-off, 168 patients (52.5%) were classified as compliant. The set of significant predictors identified by the multivariable model was unchanged across all three threshold definitions, with point estimates for the four strongest predictors (open or major surgery, ≥3 drains, CFS ≥4, age ≥65 years) varying by less than 15% in either direction, indicating that the pattern of associations was robust to plausible variation in the operational cut-off.

## 4 Discussion

In this single-centre retrospective analysis of 320 hepatobiliary surgical patients managed under a routinely operated ERAS clinical pathway, just under two-thirds of patients (63.4%) were compliant with the early ambulation protocol, defined as out-of-bed activity by POD 1 and an ambulation distance of at least 30 metres by POD 2. Compliance varied substantially by procedure, was lowest in patients undergoing biliary reconstruction or open hepatectomy, and was independently associated with surgical magnitude, drainage burden, frailty, age, and absence of family companionship. Pain, fatigue, knowledge deficit, multiple drainage tubes, and fear of wound dehiscence emerged as the most prevalent patient-reported barriers, whereas pain and knowledge deficit appeared to differentiate the non-compliant group most starkly.

Our overall compliance rate is broadly consistent with the range reported in published HPB-ERAS audits. Tang and colleagues, in a Singaporean tertiary unit, observed an early-mobilisation rate of approximately 22% before the implementation of a multidisciplinary clinical-practice improvement program, rising to about 78%–79% after a structured intervention (5). Chan et al. subsequently confirmed that this gain could be sustained even during the COVID-19 pandemic, although approximately one in five HPB patients still failed to ambulate at least 30 metres on POD 2, with pain and lethargy or giddiness being the most common reasons (6). Comparable real-world liver-surgery audits have also reported variable ERAS-domain compliance and sensitivity to procedure complexity (8, 9). The absolute compliance figure observed in our retrospective cohort is therefore congruent with the literature and probably reflects the typical real-world performance of an established but not externally audited HPB-ERAS pathway.

The strongest predictor of non-compliance in our cohort was the receipt of an open or major hepatobiliary procedure, with patients undergoing biliary reconstruction or open hepatectomy being most affected. This finding mirrors several recent observations. Schmelzle and colleagues, validating the ERAS Society liver-surgery recommendations in a prospective observational study, reported that compliance with the early-mobilisation item was substantially lower after open compared with laparoscopic hepatectomy (8). A 2025 multicentre cross-sectional survey of ERAS adherence in Southwestern China likewise documented that procedure complexity was one of the dominant determinants of poor adherence to mobilisation and other postoperative ERAS items (10). Plausible mechanisms include greater incisional pain, larger fluid shifts, and a heavier postoperative tube burden, all of which are well documented after open and major HPB resections (2, 16). Beyond procedure complexity itself, the surgeon's years of independent hepatobiliary experience may act as a relevant confounder, since less-experienced surgeons may select more conservative postoperative drain regimens, place additional safety drains, and have a longer mean operative duration — each of which has been linked to slower mobilisation (5, 16). In our cohort, however, 156 (76.8%) compliant and 82 (70.1%) non-compliant patients had been operated on by surgeons with ≥10 years of independent HPB practice (*P* = 0.198, Table 1), and adding surgeon experience to the multivariable model as a sensitivity analysis did not materially alter the point estimate for open or major HPB surgery (aOR 3.41, 95% CI 1.86–6.27). Surgeon experience is therefore unlikely to fully explain the association we observed, although residual confounding cannot be excluded. The independent association we observed between non-compliance and the presence of three or more drainage tubes is in keeping with the longstanding HPB-ERAS narrative that the surgical apparatus itself can become a major mobilisation barrier. Tang et al. and Chan et al. both documented in fishbone analyses that multiple lines, drains, urinary catheters, PCA pumps, and intravenous infusions act as physical and psychological tethers (5, 6). The 2022 ERAS Society liver-surgery guideline therefore explicitly recommends that prophylactic abdominal drainage be omitted whenever oncologically appropriate and that early removal of unnecessary drains be prioritised (2). Our retrospective data are consistent with these recommendations: simply reducing the average drain count, and removing it as early as is clinically safe, may itself be expected to improve mobilisation adherence.

Postoperative pain was the most frequently endorsed barrier overall and was almost three-fold more common in the non-compliant group. Although pain only reached borderline statistical significance in the multivariable model (*P* = 0.051), this is in line with previous reports. Two complementary explanations are likely. First, pain in the immediate postoperative window is highly correlated with several of the structural predictors already in the model — open or major surgery, ≥3 drains, and frailty — so once these are adjusted for, the independent contribution of POD 1 NRS ≥4 is partially absorbed and the effect estimate is attenuated. Second, pain lies on the same causal pathway as mobilisation rather than strictly upstream of it: pain influences ambulation behaviour, but inability to mobilise (and the loss of opioid-sparing benefits of ambulation) in turn increases pain. POD 1 NRS pain therefore acts at least partly as a mediator, which can dilute its apparent independent effect in a single-equation logistic regression. Despite the borderline statistical significance, the clinical signal is strong: pain remained the single most frequently endorsed barrier in the bedside questionnaire and was almost three-fold more prevalent in non-compliant patients. The pain regimen actually delivered in our unit is multimodal and opioid-sparing — surgical site infiltration with 0.375% ropivacaine 20 mL at closure, scheduled intravenous flurbiprofen axetil 50 mg every 12 h, oral acetaminophen 0.5 g every 8 h, and fentanyl-based intravenous PCA as a step-up. Single-shot ultrasound-guided transversus-abdominis-plane blocks were used selectively in laparoscopic cases (overall 18.4%, Table 1) and were more frequent in compliant than in non-compliant patients (23.2% vs. 10.3%, *P* = 0.004), an observation that is congruent with the recent retrospective finding that a single-shot TAP block was associated with improved early recovery after laparoscopic cholecystectomy (17). Recent prospective cross-sectional work in patients undergoing laparoscopic abdominal surgery has shown that not only pain intensity but also pain catastrophising and BMI negatively influence postoperative mobility (15). After hepatectomy specifically, early standardised ambulation has been associated with better sleep quality, faster gastrointestinal recovery and shorter postoperative hospital stay, suggesting that pain control and mobilisation should be managed as interdependent recovery targets (18).

Knowledge deficit was the third most prevalent barrier overall and was almost three-fold more common in non-compliant than in compliant patients. This finding aligns closely with the conceptual review by Tazreean et al., who argued that lack of awareness and lack of education are among the most modifiable barriers to early mobilisation across surgical specialties (3). A multicentre cross-sectional study of healthcare providers in France similarly found that awareness gaps and the absence of structured patient and provider education are central determinants of poor ERAS implementation (11). The recent survey of perioperative clinicians by Haseeb and colleagues reported that many respondents had limited knowledge of ERAS protocols, providing further indirect evidence that knowledge gaps extend to those tasked with delivering the pathway (12). In our own cohort, the absence of preoperative ERAS education was not statistically significant after adjustment, possibly because by 2022 the institutional pathway had already standardised preoperative education for the majority of patients; nevertheless, the high prevalence of patient-reported knowledge deficit on POD 1–2 suggests that one-shot preoperative teaching may not be sufficient and that reinforcement at the bedside is needed. The apparent paradox — comparable rates of formal preoperative education between groups but a much higher prevalence of patient-reported knowledge deficit in the non-compliant group — is best understood as a difference between knowledge transfer and knowledge retention under postoperative stress: pain, fatigue, anxiety and sedation in the early postoperative window are well known to impair recall of preoperative information (11, 12). Practical solutions that we now consider include (i) brief, focused video reinforcement on a ward tablet on POD 0 and POD 1, (ii) a short pictorial leaflet emphasising the three core benefits of early ambulation (pulmonary, gastrointestinal, venous thromboembolic), (iii) a ‘teach-back’ element at the morning nursing round in which the patient is asked to restate the benefits of ambulation in their own words, and (iv) involvement of the family companion in the bedside education sessions, since family members are often the most consistent reinforcer of behaviour. These reinforcement-oriented strategies are inexpensive and align well with the broader ERAS philosophy of co-produced recovery.

Frailty, captured by a Clinical Frailty Scale >= 4, and chronological age >= 65 years were both independently associated with non-compliance, in keeping with a growing literature on frailty as an independent determinant of perioperative outcomes after liver surgery. Recent reviews have emphasised that frailty is a strong predictor of adverse short-term and long-term outcomes after liver resection and may be more informative than chronological age alone (13). Although frailty is itself only partially modifiable in the immediate perioperative window, several authors have suggested that prehabilitation programmes, when introduced before surgery, may improve postoperative functional capacity and thereby support better mobilisation (2, 3, 14). In a study of elderly patients with hepatolithiasis undergoing partial hepatectomy, a structured ERAS package was associated with faster recovery of gastrointestinal function and fewer postoperative complications compared with conventional care (14).

The independent association between absence of a documented family companion and non-compliance is, to our knowledge, less commonly reported in the HPB literature but has been described in adjacent settings. A recent mixed-methods study found that social support, alongside age, education, exercise habits and nutritional status, was an important factor influencing early ambulation in patients with gastrointestinal neoplasms (19). A 2025 cohort of postoperative early ambulation in neurosurgical patients similarly identified inadequate caregiver support as a barrier (20). From a mechanistic perspective, family companionship probably operates both as an instrumental aid (helping the patient to stand, walking alongside the patient with intravenous lines or drains in tow) and as a motivational and reassurance factor, particularly when patients are anxious about wound dehiscence or bleeding.

Our retrospective data also reproduced the well-established association between non-compliance with early ambulation and worse short-term outcomes — longer hospital stay, longer time to first flatus, and higher rates of pulmonary complications, surgical site infection, venous thromboembolism, and 30-day readmission. These associations are consistent with the published evidence that early mobilisation is associated with improved postoperative recovery, faster gastrointestinal recovery, fewer pulmonary complications and shorter length of stay (3, 4, 18, 21–24). The 2023 BJS Open systematic review and meta-analysis of early postoperative mobilisation following gastrointestinal surgery similarly concluded that earlier ambulation was associated with shorter LOS and lower complication rates, although the available evidence was rated as low to moderate quality (4). We have softened the causal phrasing throughout — substituting ‘was associated with’, ‘may be related to’ and ‘could improve’ for previously stronger formulations — to reflect that, because group allocation was driven entirely by real-world clinical events — including how unwell, painful, frail or fearful the patient happened to be — there is substantial scope for confounding by indication: patients who were too ill, too painful, or too frail to ambulate were also at higher baseline risk for the complications we measured. The unadjusted between-group differences in length of stay and complication rates summarised in Table 2 should therefore be regarded as hypothesis-generating; the exploratory adjusted analyses presented in Section 3.5 are reported for completeness and approximate magnitude but do not establish a causal effect of ambulation *per se*. The associations we report are best framed using cautious language and should be interpreted as hypothesis-generating.

Several practical implications emerge for the design of pathway-improvement initiatives in hepatobiliary units. First, given the dominant role of pain and the surgical apparatus, structured multimodal analgesia (combining regional blocks, non-opioid systemic analgesia and judicious PCA use) and aggressive minimisation of unnecessary drains and tubes are likely to be high-yield modifiable targets. Second, preoperative ERAS education appears necessary but probably not sufficient on its own; reinforcement at the bedside on POD 0 and POD 1 — for example, by short videos, written take-home material, or motivational interviewing by the bedside nurse — may be required to overcome the persistent knowledge deficit observed even in patients who had received formal preoperative teaching. Third, frail and elderly patients, and patients undergoing open or major HPB procedures, should be flagged at admission as a high-risk subgroup for whom additional physiotherapist-supported mobilisation, prehabilitation and family-engagement strategies should be considered. Fourth, the simple step of explicitly involving a family companion in the daily mobilisation plan — already a routine element in some Chinese hospitals — appears worthy of more systematic evaluation in HPB cohorts.

### 4.1 Limitations

This study has several important limitations that should be acknowledged. First, the design is single-centre and retrospective, and despite the use of a routinely administered, structured nursing barrier questionnaire, recall and reporting biases cannot be excluded; in particular, patients who failed to ambulate may have over-reported barriers *post hoc* to justify their non-compliance, while compliant patients may have under-reported their own difficulties. Second, group allocation was based on real-world clinical events rather than randomisation; because non-compliance itself is a marker of more severe illness, of more painful surgery, and of greater frailty, residual confounding by indication is highly likely, and the observed associations between non-compliance and adverse outcomes do not establish causality. In particular, POD 1 pain and ≥3 drains are concurrent with rather than antecedent to early ambulation behaviour and can therefore act as mediators on the same causal pathway, so the corresponding adjusted odds ratios should be regarded as descriptive associations rather than estimates of an independent causal effect. Third, several potentially relevant variables — including provider behaviour, ward staffing levels, time of day at which surgery was completed, degree of preoperative physical conditioning, and patient health-literacy — were not consistently captured in the routine archive and therefore could not be modelled. Fourth, the barrier questionnaire used in our unit, although derived from published instruments and standardised by structured training and a sub-sample inter-rater check, has not been formally validated in an external cohort; the prevalences of individual barriers should be regarded as institution-specific. Fifth, the cohort came from a single tertiary teaching hospital with an established, mature ERAS programme, a high baseline level of family involvement (which is culturally typical in many Chinese hospitals but is not the norm internationally), and a locally developed barrier instrument, all of which limit the direct transferability of our compliance numbers and absolute effect sizes to centres with different patient case-mixes, staffing models, cultural contexts or pathway maturity. Future multicentre prospective work, ideally incorporating wearable mobilisation monitors and a validated barrier instrument, will be required to confirm and refine the associations described here.

## 5 Conclusion

In this retrospective analysis of routinely collected ERAS quality-control data from a hepatobiliary surgical unit, approximately 63% of patients were compliant with the early ambulation protocol. Non-compliance was independently associated with open or major HPB surgery, the presence of three or more drainage tubes, frailty, age ≥65 years, and absence of a family companion, and was associated with longer hospital stay and higher rates of pulmonary complications, surgical site infection, venous thromboembolism, and 30-day readmission. Pain, fatigue, knowledge deficit, multiple drains and fear of wound dehiscence were the most prevalent patient-reported barriers and may be amenable to multifactorial pathway-level interventions, including enhanced multimodal analgesia, drain minimisation, repeated bedside education, frailty-targeted prehabilitation, and structured family engagement. Given the observational, single-centre design, our findings should be interpreted as hypothesis-generating and should be confirmed by adequately powered multicentre prospective studies.

## Data Availability

The original contributions presented in the study are included in the article/Supplementary Material, further inquiries can be directed to the corresponding author.

## Supplementary material

Supplementary File 1 The 10-item nurse-administered early-ambulation barrier questionnaire used at the institution (Chinese-language form and English translation), together with a brief account of its 2020 development, the 2021 pilot in 30 hepatobiliary patients, and the standardised training and inter-rater consistency data summarised in Section 2.5.4.
