# Supplementary material for: Compliance with early postoperative ambulation and Its associated barriers in hepatobiliary surgery patients within an enhanced recovery after surgery framework
Source: Front Surg. 2026 Jun 24;13:1874831. doi: 10.3389/fsurg.2026.1874831 (PMC13341614; doi:10.3389/fsurg.2026.1874831)
Supplement: Supplementary File 1 — The 10-item nurse-administered early-ambulation barrier questionnaire used at the institution (Chinese-language form and English translation), together with a brief account of its 2020 development, the 2021 pilot in 30 hepatobiliary patients, and the standardised training and inter-rater consistency data summarised in Section 2.5.4. [file Datasheet1.pdf]

Supplementary File 1

Nurse-Administered Early-Ambulation Barrier Questionnaire (10-item)

1. Background and development

This 10-item barrier questionnaire has been used as part of routine Enhanced Recovery After Surgery (ERAS) quality control in the Department of Hepatobiliary Surgery of the Affiliated Hospital of Jiangnan University since mid-2021. It was developed by a working group consisting of senior ward nurses, two ERAS coordinators and one surgical methodologist, on the basis of the published hepatopancreatobiliary ERAS and gastrointestinal-surgery barrier literature and the conceptual barrier framework proposed by Tazreean and colleagues. An initial 13-item draft was piloted on 30 consecutive hepatobiliary surgical patients during the first quarter of 2021; based on bedside feedback and review by the working group, three semantically overlapping items were removed and three items were re-worded for clarity, producing the final 10-item version that has been used unchanged across the unit since mid-2021.

2. Administration and training

The questionnaire is administered by the bedside nurse on postoperative day (POD) 1 and POD 2 to every patient enrolled on the institutional ERAS pathway, irrespective of whether the day's prescribed ambulation was eventually achieved. For each item, the nurse asks whether the listed factor prevented or limited the prescribed ambulation that day, and records a yes/no response in the patient's electronic nursing record. Bedside nurses receive standardised training on questionnaire administration during their unit induction and at annual refresher sessions. In a quality-control sub-sample of 40 paired patient-nurse administrations, the inter-rater consistency between two independent nurses for each item was substantial (Cohen's $\kappa = 0.81$). For the present analysis, a barrier was considered "present" if it had been endorsed on at least one of POD 1 or POD 2.

3. Items (English translation)

Item	Domain	Question (English)
1	Pain	Did postoperative pain (NRS $\geq$ 4 at the time of planned ambulation)

		prevent or limit the prescribed ambulation today?
2	Fatigue / weakness	Did generalised fatigue or weakness prevent or limit the prescribed ambulation today?
3	Drainage burden	Did the presence of multiple drainage tubes (≥ 3 tubes/lines tethered to you) prevent or limit the prescribed ambulation today?
4	Fear	Did fear of wound dehiscence or postoperative bleeding prevent or limit the prescribed ambulation today?
5	Dizziness	Did dizziness on standing prevent or limit the prescribed ambulation today?
6	Nausea / vomiting	Did nausea or vomiting prevent or limit the prescribed ambulation today?
7	IV / PCA tether	Did the intravenous infusion line or PCA pump (perceived as a tether) prevent or limit the prescribed ambulation today?
8	Knowledge deficit	Were you unaware of, or unsure about, the benefit of early ambulation, in a way that prevented or limited the prescribed ambulation today?
9	Anxiety / depression	Did anxiety or depressive symptoms prevent or limit the prescribed ambulation today?
10	Family companionship	Did inadequate family companionship at the time of planned mobilisation prevent or limit the prescribed ambulation today?

4. Items (Chinese-language form used at the bedside)

项目	维度	问题（中文）
1	疼痛	今日计划下床活动时，是否因术后疼痛（NRS ≥ 4 分）而无法或不能完成医嘱下床活动？
2	疲乏 / 虚弱	今日是否因全身乏力或虚弱而无法或不能完成医嘱下床活动？
3	管路负担	今日是否因身上同时连有 ≥ 3 根管道（引流管、尿管、补液管、镇痛泵等）而无法或不能完成医嘱下床活动？
4	恐惧	今日是否因担心伤口裂开或术后出血而无法或不能完成医嘱下床活动？
5	头晕	今日是否因站立时头晕而无法或不能完成医嘱下床活动？

6	恶心 / 呕吐	今日是否因恶心或呕吐而无法或不能完成医嘱下床活动？
7	输液 / 镇痛泵牵绊	今日是否因输液管或镇痛泵牵绊而无法或不能完成医嘱下床活动？
8	知识缺乏	您是否因不了解或不确定早期下床活动的好处而无法或不能完成今日医嘱的下床活动？
9	焦虑 / 抑郁	今日是否因焦虑或情绪低落而无法或不能完成医嘱下床活动？
10	家属陪护不足	今日计划下床活动时，是否因缺乏家属陪护而无法或不能完成医嘱下床活动？

5. Scoring and data handling

Each item is scored dichotomously (1 = yes, prevented/limited the prescribed ambulation that day; 0 = no). Daily responses are entered into the institutional ERAS quality-control dashboard. For the present analysis, a barrier was treated as present at the patient level if it had been endorsed on at least one of POD 1 or POD 2. No total or summative "barrier score" was computed; each barrier was analysed individually as a binary variable. By construction, a patient who fully achieves the prescribed ambulation on both days may still endorse one or more barriers ("limited but completed"), and this design therefore captures concurrent perceived barriers in both compliant and non-compliant patients rather than only retrospective justifications in patients who failed to mobilise.

6. Limitations of the instrument

As stated in the main manuscript (Section 4.1), although the questionnaire was systematically developed from published barrier literature, piloted, refined, and supported by standardised nurse training and a small inter-rater check (Cohen's $\kappa = 0.81$), it has not undergone formal psychometric validation (factor analysis, test–retest reliability, construct validity testing) in an independent external cohort. The reported prevalences of individual barriers should therefore be regarded as institution-specific and as exploratory rather than as definitive epidemiological estimates of barriers to early ambulation in HPB surgical patients more generally.
